# Investigating the confinement effects on masonry behavior based on friction interface

**DOI:** 10.1038/s41598-024-62237-2

**Published:** 2024-06-06

**Authors:** Hafiz Asfandyar Ahmed, Waqas Arshad Tanoli

**Affiliations:** 1 Department of Civil Engineering, University of Engineering & Technology, Peshawar, Pakistan; 2 Department of Civil and Environmental Engineering, College of Engineering, King Faisal University (KFU), P.O. Box 380, 31982 Al-Hofuf, Al Ahsa Saudi Arabia

**Keywords:** Numerical modeling, Friction interface model, Confined masonry, Unconfined masonry, Simplified micro modeling, Engineering, Civil engineering

## Abstract

As a material masonry is anisotropic in nature as it is constituted of various components that differ each other in many ways. Though, the ideas of modeling of concrete are also valid for masonry, still there are difficulties in determining the exact behavior of masonry. The micro modeling (simplified) approach has been adopted in this study for modeling masonry samples subjected to various nature of loadings. The brick masonry units were modeled as discrete elements and linked together by interface element. The coulomb friction model was used to describe the interface element as mortar joint. Damage was assumed to be governed by the compressive strength of both the constituents i.e., mortar and brick. The mechanical properties of the masonry samples were determined by means of variety of tests and those properties then served as input parameters in the constitutive model. The results clearly indicate that numerical model predicted the behavior of the experimentally tested samples, in compression as well as tension prims for both unconfined and confined cases. The proposed model is suitable for modeling any type of brick masonry walls.

## Introduction

Masonry as a structural material is almost the most extensively used construction practice because of its intrinsic advantages that comprise of durability, economy, aesthetics, ease of construction, low maintenance, fire resistance, reasonable thermal and sound insulation among others^1^. Brick units are made at local level throughout the world using various materials and dimensions and different production procedures, are being employed for masonry construction^2^. While the use of masonry in construction is practiced for long, the response analysis of masonry construction has been investigated in approximately last four decades or so^3^ and still needs a complete understanding. Masonry exhibits inelastic, non-homogeneous and anisotropic response as it is made of two different materials. In published literature, strong brick-weak mortar and weak brick-strong mortar masonry have been reported. For instance, Singh and Munjal^4^, Ravula et al.^5^, Balasubramanian et al.^6^ and Nagarajan et al.^7^ reported a relatively stiffer mortar as compared to brick. However, others refer to stronger unit than mortar^8^. Mohamed et al.^9^ stated that mortar is mainly the reason for masonry’s non-linear response. Thus, brick- and mortar-strength and their relative volume influences the masonry performance. The analytical models specified by MSJC^10^ and Euro code 6^11^ and by other researchers e.g., Engesser^12^, Bröcker^13^, Mann^14^, Hendry and Malek^15^, Dayaratnam^16^, Bennet et al.^17^, Dymiotis and Gutlederer^18^, Gumaste et al.^8^, Kaushik et al.^19^, Christy et al.^20^, Garzón-Roca et al.^21^, Lumantarna et al.^22^, and Kumavat^23^ largely comprise of the strength of brick and mortar. Researchers’ pay a lot of attention to the novel procedures of analyzing the masonry^24–33^. These techniques are generally grounded on development of numerical approaches validated against some experimental results. The Non-Linear Finite Element Method (FEM) is an influential tool to model a structure that have large number of elements. It can be achieved by transforming the original assembly into pure finite elements with assessed properties like elastic modulus, tensile and compressive strength etc. Basically, the FEM modeling of masonry is divided into two groups i.e., heterogeneous and homogeneous. In heterogeneous approach, both units and mortar are modeled discretely, while in the homogeneous one the units and mortar are supposed to be merged into a single compound material bearing average properties. Although homogeneous method is conventionally used for modeling of masonry structures, heterogonous ones are more illustrative^34^. Modeling masonry with heterogeneous approach can enhance the outputs in comparison to the homogenous method. Test data^35–37^ revealed that cracking of masonry is due to the de-bonding of units and mortar joints, however, the response of post-cracking till collapse is characterized by the unit-mortar interface.

In general, to predict the masonry behavior in a realistic way a model necessitates interface elements. A likely option can be adopting an interface element as joint and appropriate model for the units. Hence, almost all the vital features influencing the masonry response are considered in this case, and a general-purpose FE software can be used for solving the numerical model. Hence, a simplified micro modeling (meso-modeling) approach taking units as discrete elements and joined together by interface elements, is adopted for modeling in this study. Interface elements were used using cohesive surface-based behavior to connect the brick units. This model comprises of material properties such as stress–strain, response, and elastic/inelastic criteria.

### Micro and macro modeling

**Figure 1:**
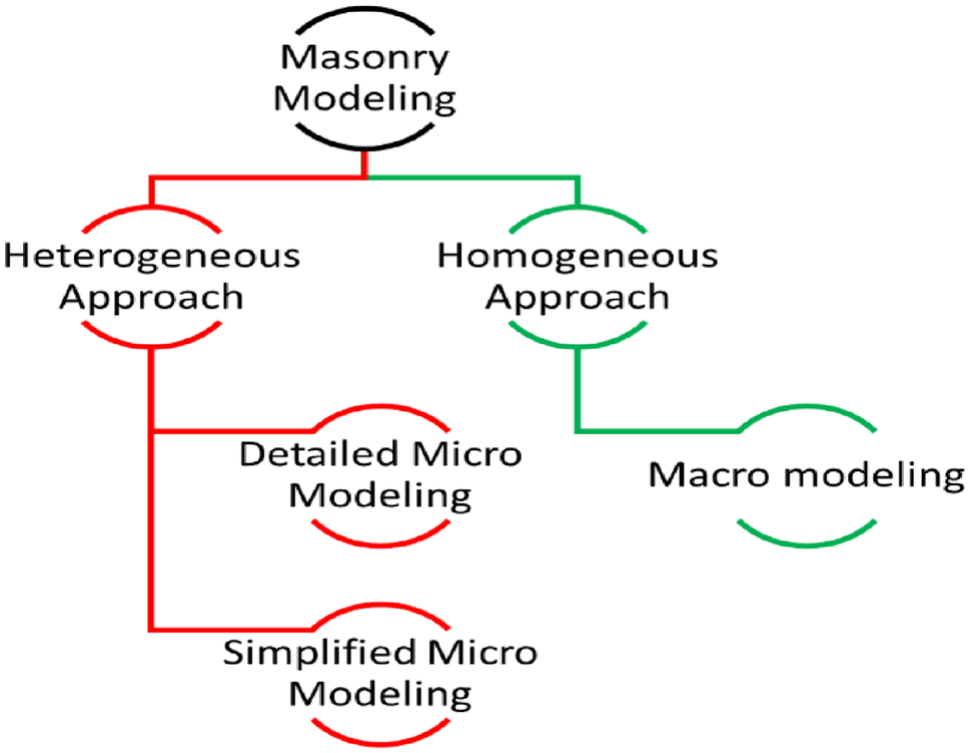
Modeling strategies of masonry.

Masonry is an an-isotropic and heterogeneous material because of different constituents for example mortar joint, brick unit, and existence of head and bed joint courses. Various approaches of modeling the masonry are presented in Fig. 1 and are briefly discussed here.

Macro modeling: It is established on the homogeneous approach and offers an estimated response somehow for elementary design and analysis. In this method, masonry is taken as a composite material and it is employed to assess the general behavior of the assembly^38^. This technique was adopted for big models in which the brick units and joints are smeared into orthotropic material. But apparently masonry is anisotropic, so such model will not project the local response of the masonry structure.

Micro modeling: In this technique, brick units and mortar joint are considered as continuum elements while the unit/mortar interface as intermittent elements. Various parameters like elastic modulus, Poisson’s ratio, and other inelastic properties, are accounted for both unit and mortar in this method. Though this method is more genuine and determines the local response of masonry accurately, but it raises a question of complexity by modeling all the masonry constituent and thus making this method extravagant and time consuming. As a result, a simplified micro modeling (also called meso modeling approach) has been developed and most of the research work on masonry modeling, including but not limited to Shing et al.^25^, Berto et al.^27^, Milani^28^, Stavridis and Shing^32^ and La Mendola et al.^39^ have adopted modeling technique.

**Figure 2:**
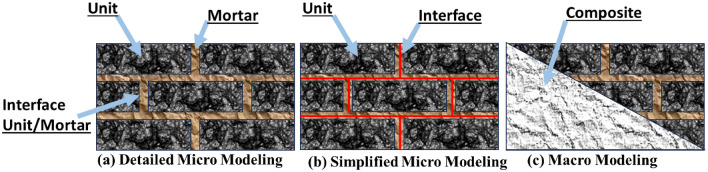
Suggested modeling strategies, Lourenco^26^.

In this method, both head and bed joints are clutched into an interface element and the brick units are expanded to the half of the thickness of the joints. Shing et al.^40^ have adopted discrete crack method for masonry walls as a simplified micro model. The primary source of crack was defined through interface elements; however, the secondary cracks were considered into the units by smeared crack method. This method effectively estimated the response of masonry samples in comparison with the experimental test results. Lourenco and Rots^26^ employed an interface element to study the unreinforced masonry assemblies. Many failure patterns were considered, and a novel approach was established (details in Fig. 2) for masonry modeling. In this method, the joints are assumed to be the vulnerable component and demonstrated by an elasto plastic element. It was revealed from the results that the suggested model had been able to replicate the experimentally tested behavior deprived of any numerical complications. Furthermore, the prescribed model can predict the cracks within the units. Apprehending this kind of cracking was a great success in modeling the masonry.

Shing and Cao^41^ worked out two distinct kinds of elements for predicting the response of masonry walls; brick units were modeled as smeared while mortar joints were as interface elements. Results presented that the failure mode was well predicted in the model. Homogenization method is another technique in modeling of masonry which acts like a midway amongst micro and macro element modeling approaches. This method is adopted primarily by Milani et al.^42^, Casolo and Milani^43^ and Milani^30,31^, is a suitable approach for a non-homogeneous material comprising of intermittent units. In this formulation the operator only needs relatively smaller number of parameters by evading separate modeling the joints and units present in the assembly. Employing this approach causes a reduction of time and efforts in modeling process and computational cost of model.

Masonry is a non-homogeneous, anisotropic and in-elastic in nature because it is combination of two different components (brick and mortar, both are mixtures of several other materials) with varying mechanical properties in each orthogonal direction. A lot of research is still going on to completely understand the behavior of masonry in a more detailed way. There are many areas that needs to be explored in-depth for better understanding and description of masonry response. For example, very limited research is available in the published literature, that addresses the effect of vertical loads on masonry in combination with the confinement effect in in-plane direction, particularly at material level. It is very important to understand the interaction of vertical loads with the confinement effect in uni-directional manner, since in masonry construction (either in confined masonry or infill walls) such cases exist. And understanding them well, will help to further optimize their design for practical applications. In addition to that, the impact of boundary conditions and most particularly that of confining element type in in-plane direction is also not considerably investigated at element level. Comprehensive research that focuses on the effect of boundary conditions, like edge restrains or support conditions on the global response and failure pattern of masonry samples that are confined in in-plane direction, can provide effective understanding of the masonry behavior. This research work aims to investigate the research gaps just identified, to contribute in better understanding of the masonry response under vertical/gravity load when confined in one direction (i.e., in-plane), that will lead to the improvement of strategies that will enhance the structural performance of masonry.

### Research objective and methods

The objective of this research work is to evaluate the effect of confining plates on the clay brick masonry response in axial compression and tension and in diagonal compression. To achieve the objective, a group of 25 prisms was constructed, instrumented, and tested. Numerical models are generated to predict the response of actual tested prisms with high accuracy and low computation time. The numerical models are then calibrated against the experimental results and in-depth analysis is performed to address various parameters that influences the masonry performance and recommendations are made to appropriate performance limit states. Ongoing research currently at UET Peshawar trails the work presented in this paper with the objective of evaluating the impact of the plates on reinforced masonry samples both experimentally and numerically.

## Experimental program

### Mechanical properties of brick and mortar

Regular solid clay brick units with nominal dimensions of 229 × 114 × 76 mm (9 × 4.5 × 3 in), as per ASTM C90^44^ standards, were employed in this study. The modulus of elasticity and compressive strength were obtained from simple compression test, while tensile strength and modulus of rupture were obtained by 3-point bending test of brick. Mortar cubes 51 × 51 × 51 mm (2 × 2 × 2 in) were tested to estimate the compressive strength, which comes out to be 5.63 MPa (816 psi). Cement to sand proportion by volume was 1:5 as per ASTM C270^45^. Table 1 presents the details of tests conducted to determine the mechanical properties of brick units and mortar. f_b_ and f_m_ denotes the compressive strength of brick unit and mortar cube respectively, f_t_ denotes the tensile strength of brick unit, E_b_ and E_m_ denotes the elastic modulus of brick unit and mortar cube respectively. The mean value for given parameters of the tested samples, were employed in the modeling procedure.

**Table 1:** Mechanical properties of brick units and mortar joints.

S. no.	Brick			Mortar	
	f_b_ (MPa)	E_b_ (GPa)	f_t_ (MPa)	f_m_ (MPa)	E_m_ (GPa)
1	16.80	25.67	1.70	4.13	7.6
2	13.02	32.57	1.32	5.13	11.9
3	12.82	25.41	1.30	5.60	11.1
4	13.86	26.48	1.41	6.80	11.7
5	15.07	22.85	1.53	7.10	10.8
6	12.35	12.32	1.25	5.64	6.9
7	14.67	23.13	1.49	5.37	8.5
8	15.66	22.36	1.59	4.98	7.1
9	12.53	18.04	1.27	5.78	8.3
10	17.22	27.66	1.75	5.77	9.3
Minimum value	12.35	12.32	1.25	4.13	6.9
Maximum value	17.22	32.57	1.75	7.10	11.9
Mean	14.4	23.65	1.46	5.63	9.3
COV	0.12	0.23	0.12	0.15	0.21

### Interface failure characterization

#### Cohesion and internal friction of interface

**Figure 3:**
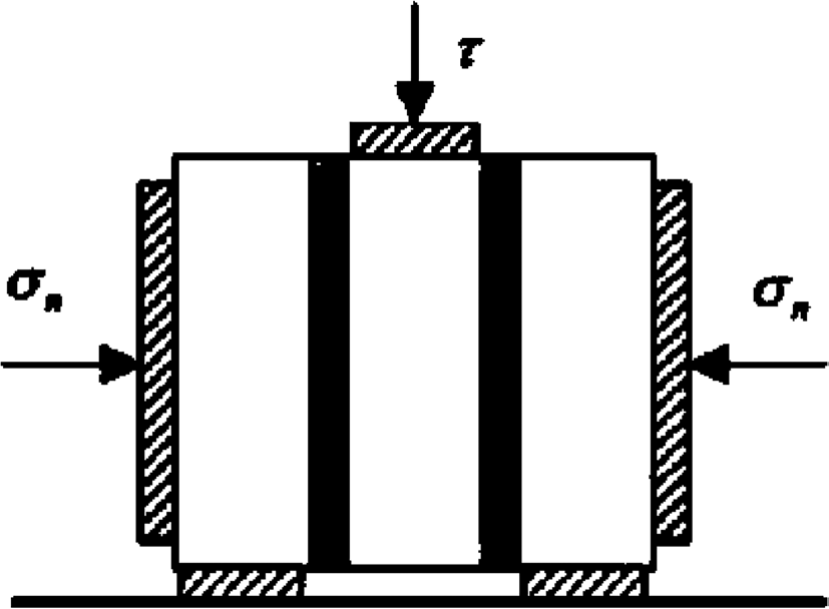
Shear test setup for determination of c–φ.

In addition to the mechanical properties of unit and mortar, another most important parameter particularly used in this study was the determination of cohesion (c) and internal friction (φ) that is usually determined from triplet test on masonry sample. ASTM specifications do not propose any measures for calculating the c and µ from the triplet test. To calculate these factors for masonry shear strength EN-1052-3^46^, standards were adopted to implement triplet test (see Fig. 3). A total of fourteen triplets were tested. Test on five samples were performed without any pre-compression load however three triplet samples each were tested under pre-compression load of 0.78 tons, 1.56 tons and 2.34 tons respectively.

The shear strength of the brick/mortar interface typically depends on the normal stress applied to the interface. This friction type behavior is classically described by the Mohr–Coulomb yield function by Eq. (1) as:

1 $$f\left( {\tau , \sigma_{n} , \phi } \right) = \left| \tau \right| - c + \sigma_{n} \tan \phi$$

where τ is the shear stress, σ_n_ is the normal stress (negative in compression), c is the cohesion of the material and φ is the internal angle of friction (Fig. 15).

Determining cohesion and the internal friction angle requires the measurement of normal and shear stresses until failure. The shear strength was determined using Eq. (2) by Popov and Balan^47^.

2 $$Shear Strength = \frac{Shear \;force\; that \;produces\; sliding }{{2 \times Shear Area}}$$

**Figure 4:**
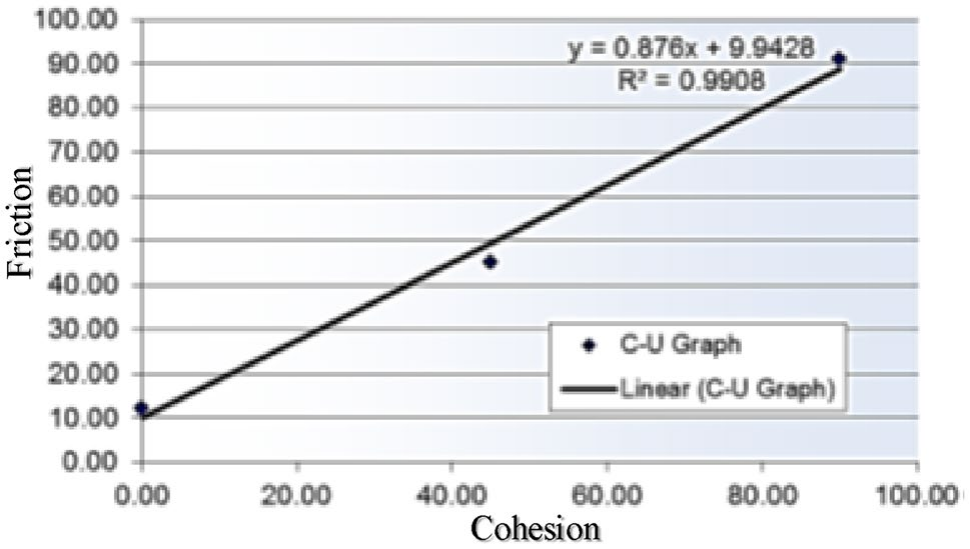
Friction and cohesion data.

The average values of both the parameters (c and φ) are given in Table 2. Though empirical equations developed by^47^ were adopted to calculate the Cohesion c and Friction Coefficient µ (tanφ) as presented in the Fig. 4.

**Table 2:** Properties of interface determined from triplet test.

Parameter	Cohesion (c)	φ
Interface	0.52	0.33

#### Tensile strength of interface

The bond strength in tension of the interface was determined by duplet testing (masonry prisms) in simple tension. The strength was evaluated by dividing the total force over the area of the sample. In this case, the mean tensile strength estimated was 0.4 MPa.

### Testing of masonry prisms

Masonry prisms with various configurations were made to evaluate various physical and mechanical parameters under unconfined compression (UC), confined compression (CC), unconfined tension (UT), confined tension (CT) and diagonal compression (DC) testing. Thirty samples were tested for each specimen to consider the variability. The results of these tests reveal the distinct response difference (strength, failure mode and deformation capacity) between different testing methods of masonry (e.g., confined vs unconfined). Cement mortar of 10 mm thickness was employed for making the test specimens.

#### Compression testing

The main objectives of this type of testing were to evaluate the confinement effect on (1) final strength, (2) strain, and (3) stress–strain relationship of brick masonry in compression. To achieve these objectives, 10 prisms were formed and tested. Steel plates were employed in this study for the application of vertical load as well as lateral confinement (in the case of confined samples). The confining stresses were applied (through nut and bolt arrangement) enough to keep the lateral plates intact while the vertical load was applied, so that the prism does not experience any deformation in the lateral in-plane direction. The special confinement plates adopted were solid steel plates with 38 mm (1.5-in) thickness.

Prisms with dimension of 400 mm width and 450 mm height were built in English bond pattern and tested using ASTM C1314^48^. Same prism configuration was adopted for both confined and unconfined tests. Axial strain was computed by means of LVDTs. Vertical compressive load was putted through UTM under displacement control environment. Steel plates were used at both top and bottom of the prism for the equal distribution of axial load to the top layer of the prism. The same loading configuration was used in both confined and unconfined testing setups.

##### Unconfined compression (UC)

**Figure 5:**
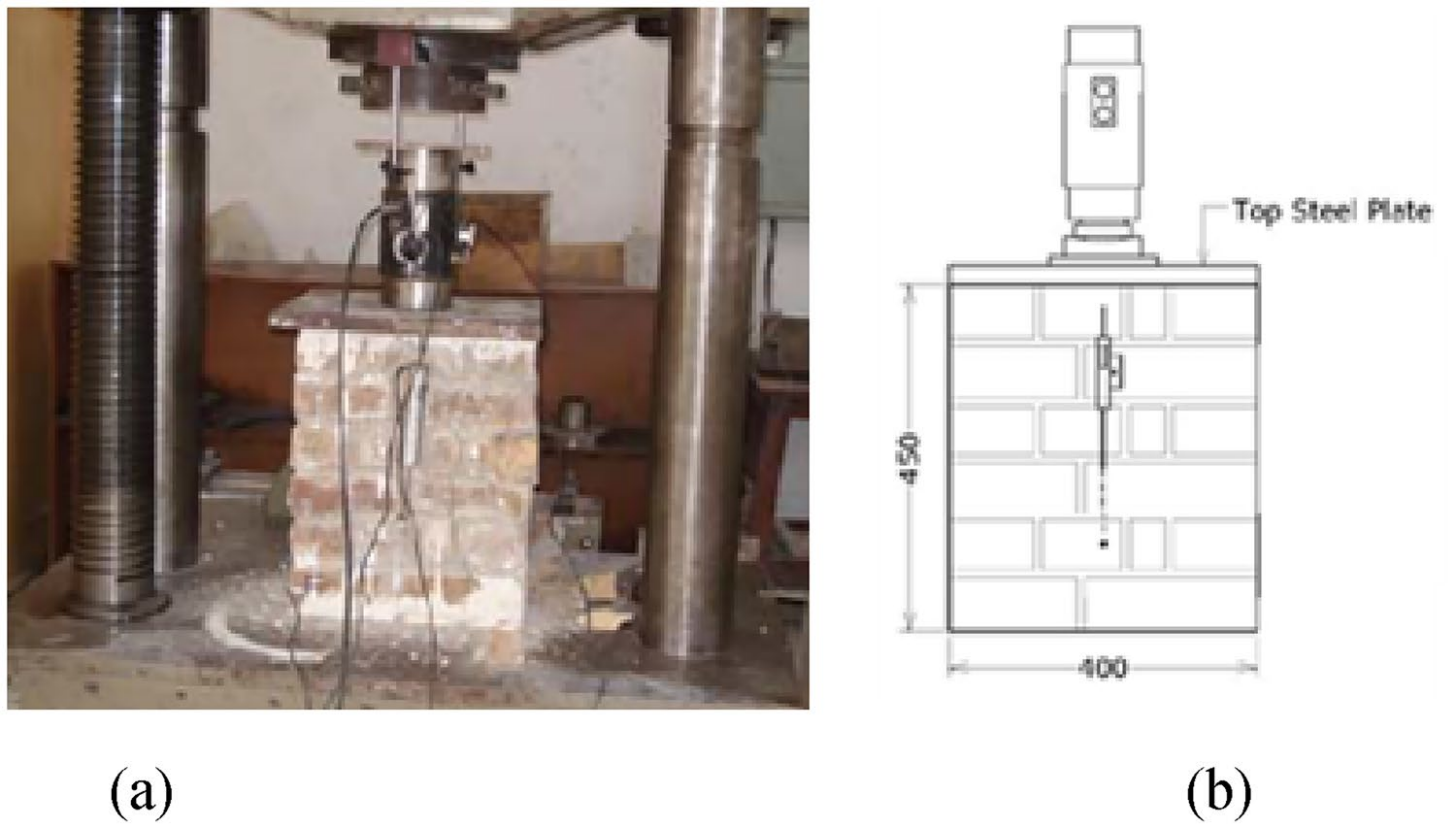
(**a**) UC Test, (**b**) dimensions and instrumentations of specimen.

The masonry compressive strength has been customarily considered as the only appropriate mechanical property of material, nonetheless up to the modern induction of numerical procedures. It is normally thought that the actual compressive strength (uniaxial) of masonry can be taken from RILEM test^49^, demonstrated in Fig. 5. The sample in this test is though fairly big as compared to the standard concrete cube or cylinder sample. Failure of the unconfined prism was governed by tensile splitting along the vertical direction, originated at the central web and propagating to the bottom and top layers of the prism.

##### Confined compression (CC)

Masonry walls are preferable designed for in-plane loads and therefore are subjected to biaxial stress, such as infill walls, etc. To calculate their complete response, understanding of the different masonry attributes like deformation and failure subjected to biaxial stress states is needed. The complete masonry behavior under biaxial state of stress (i.e., CC) cannot be fully portrayed from the uniaxial loading behavior (i.e., UC). The effect of the CC is studied to the maximum stress to give the strength envelope (biaxial), that cannot be defined merely as principal stresses due to the fact that masonry is an-isotropic in nature. The most comprehensive set of experimental data under biaxial loading was done by Page^50,51^.

In confined/biaxial compression, damage normally took place by piercing of the assembly near mid height, corresponding to the open surface, irrespective of the direction of the principal stresses. The upsurge of the strength under CC can be attributed to increased friction between units and mortar as well as internal friction within the joints.

**Figure 6:**
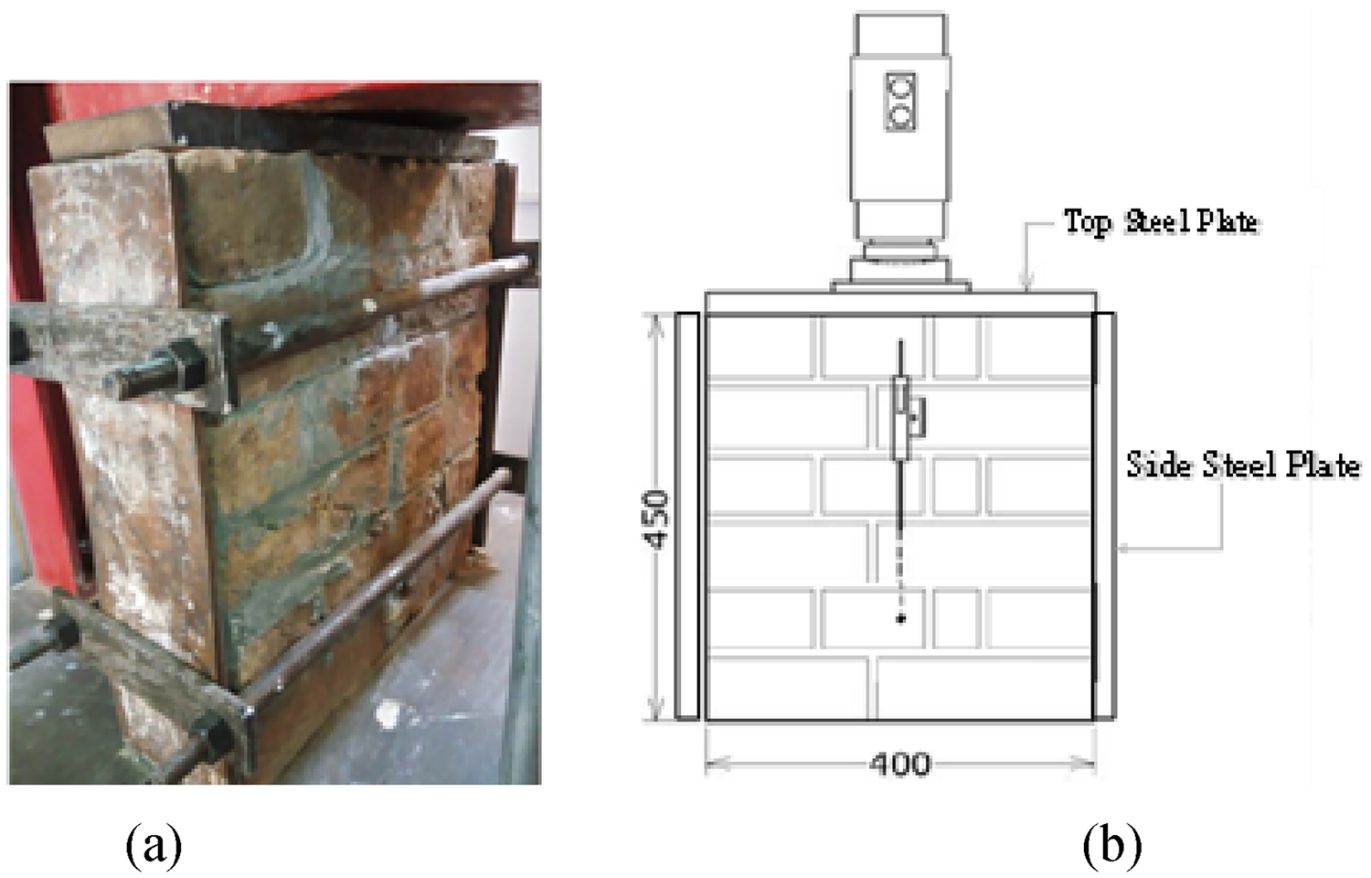
(**a**) CC test, (**b**) dimensions and instrumentations of specimen.

Steel plates were used to apply the lateral confinement effect by arrangement of nut and bolts (Fig. 6). The plates were joined together with a state of stress similar to prestressing phenomena, as already discussed in section “Mechanical properties of brick and mortar”, after which the axial compressive stress was applied to the prism. Table 3 Presents the test results of unconfined and confined prisms in compression. The compressive strength of the confined prisms is higher as compared to the unconfined prisms primarily due to the fact that lateral confinement provided resistance against the tensile splitting of the prism (in the lateral in-plane direction) and therefore the ultimate strength of the masonry increases considerably. The ultimate strength of confined sample is almost double to that of unconfined sample. Page^50,51^ tested 360 mm square prisms with the half-scale brick units used at various angle and under various loading conditions. It was concluded that when lateral load was applied perpendicular to the bed joint, a considerable enhancement in vertical load was noted. However, when the lateral load was applied parallel to the bed joint, the vertical load capacity enhanced but not to a large extent. However, since the biaxial loading was applied in these studies, the boundary conditions of the said studies does not match exactly with current study, as in the current study, the confinement plates were used to restrain the lateral in-plane displacement and not to apply a continuous lateral load. In another study, Ferretti et al.^52^ conducted uniaxial and biaxial tests on AAC masonry samples of size 625 × 750 mm and concluded that the strength of confined sample is considerably similar to the unconfined one. However, it can be attributed to the slenderness ratio of the sample, since the size of specimen in current study is 400 × 450 mm as compared to^52^. Therefore, by comparing the results of these two studies, it can be stated that the effect of specimen slenderness (both in horizontal and vertical side) plays an important role in defining the behavior masonry and lateral confinement does not have a considerable effect after a threshold value. And this threshold value needs to be identified to consider the effect of lateral confinement on vertical load carrying capacity of the specimen.

**Table 3:** Compressive strength of confined and unconfined specimens.

S. no.	Specimen type	Strength (MPa)	Average	Standard deviation
1	Unconfined	4.85	4.15	0.63
2		4.68		
3		4.10		
4		3.79		
5		3.31		
1	Confined	8.85	8.41	0.28
2		8.54		
3		8.23		
4		8.15		
5		8.30		

#### Tensile behavior of confined and unconfined masonry

**Figure 7:**
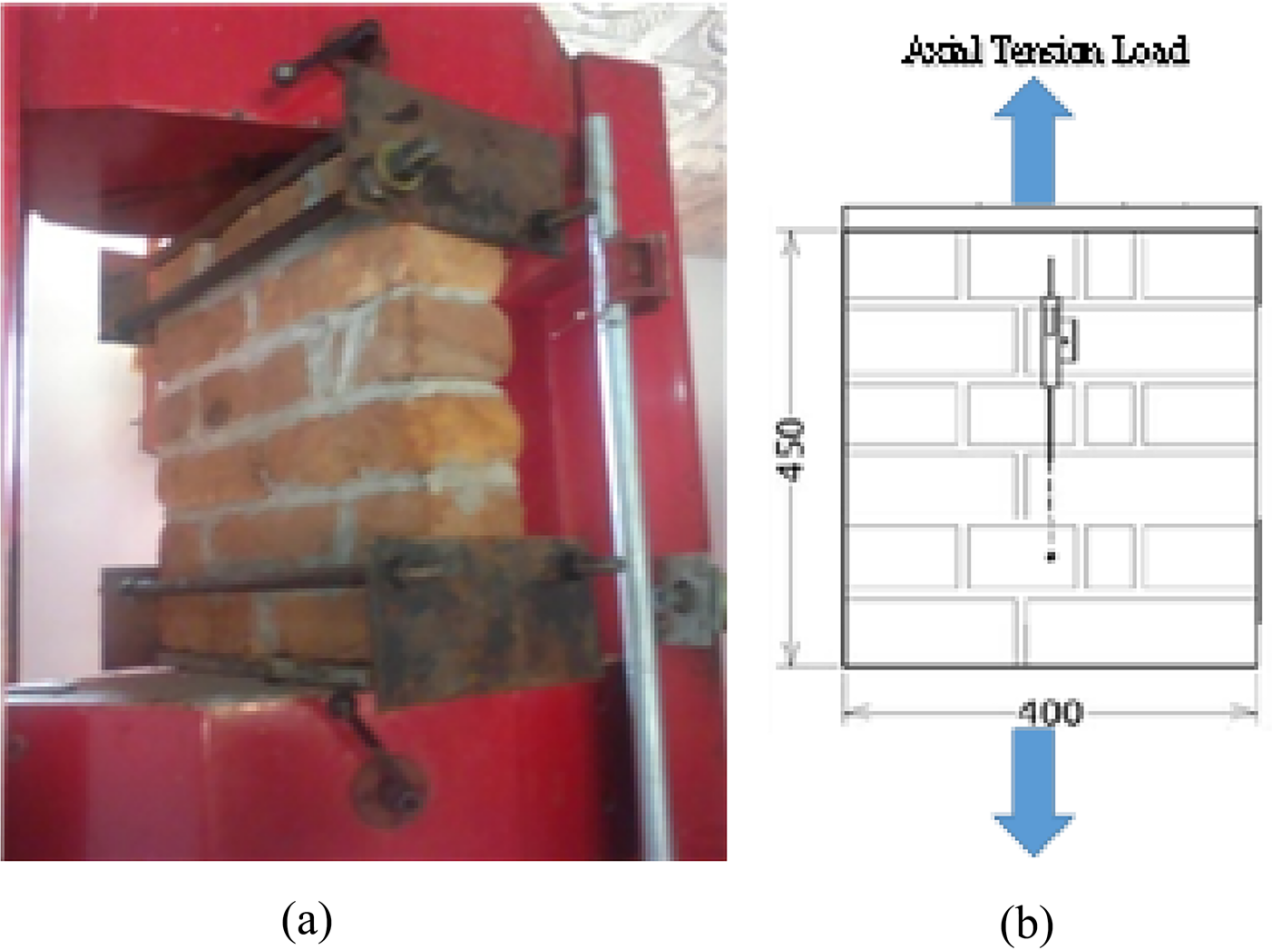
(**a**) UT test, (**b**) dimensions and Instrumentations of specimen.

**Figure 8:**
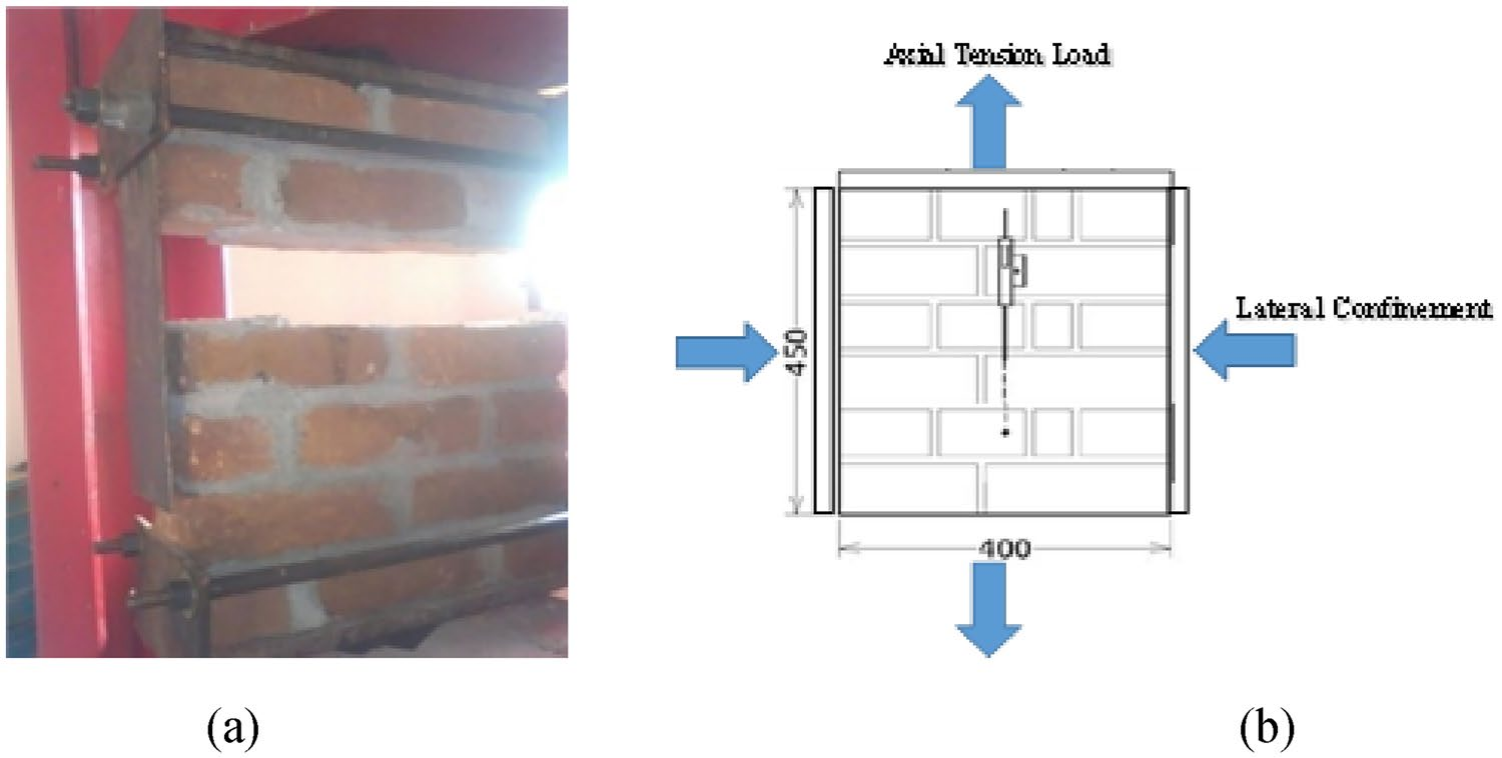
(**a**) CT test, (**b**) dimensions and Instrumentations of specimen.

This type of testing was designed to evaluate the confinement effect on tensile strength normal to the bed joint of brick masonry prisms. 10 clay brick masonry prisms with dimension of 400 mm width and 450 mm height were formed and tested in direct tension. Steel plates were employed in this study for the application of tensile load (Fig. 7) as well as lateral confinement (in the cased of confined samples). The special confinement plates adopted were solid steel plates with 38 mm (1.5-in) thickness. The confinement arrangement can be seen in Fig. 8. All other arrangements were similar as discussed in the case of compression loading test apart from the loading. Steel plates were fixed at both the bottom and top of the prism mechanically, and then the axial tension was applied on it. The test results of unconfined and confined prisms in tension are presented in Table 4.

**Table 4:** Tensile strength of confined and unconfined specimens.

S. no.	Specimen type	Strength (MPa)	Average	Standard deviation
1	Unconfined	0.23	0.22	0.027
2		0.25		
3		0.23		
4		0.18		
5		0.20		
1	Confined	0.23	0.20	0.024
2		0.21		
3		0.19		
4		0.17		
5		0.18		

##### Unconfined tension (UT)

For masonry in tension, the usual mode of failure is due to the failure of the interface amid the unit and joint. Roughly. the masonry strength in tension can be approximated to bond strength of unit mortar interface. However, in some cases the unit tensile strength also governs, when the units have low strength in comparison to the interface bond strength that may cause the units to fail first.

For UT, failure was done by cracking and sliding of bed joints. The effect of the lateral tension on the tensile strength is not identified since no tests were performed for bi-axial tension.

##### Confined tension (CT)

A more vital methodology to study the masonry strength (in shear) is based on understanding the masonry behavior subjected to biaxial state of stress, considering the application of stresses in direction relative to the bed joint. Stress was applied perpendicular to the bed joint. Generally, from the tests, mostly nonlinear response is due to the separation of unit layer from the subsequent layer along the interfaces at the mortar joints. These interfaces are the planes of weakness. Under confined-compression, tangent modulus varies by increasing the load till failure. However, in confined-tension, masonry fails in elastic manner at much low level of load; thus, masonry is linear elastic-brittle material when one of the applied stresses is tension.

It can be argued that lateral compression/confinement in masonry decreases its tensile strength due to the induced damage, by joints/interface’s micro-slipping and units’ micro-cracking. In the CT test, failure take place by joints sliding and/or cracking or in a mixed way, including both units and joints cracking/sliding.

#### Diagonal compression test (DC)

**Figure 9:**
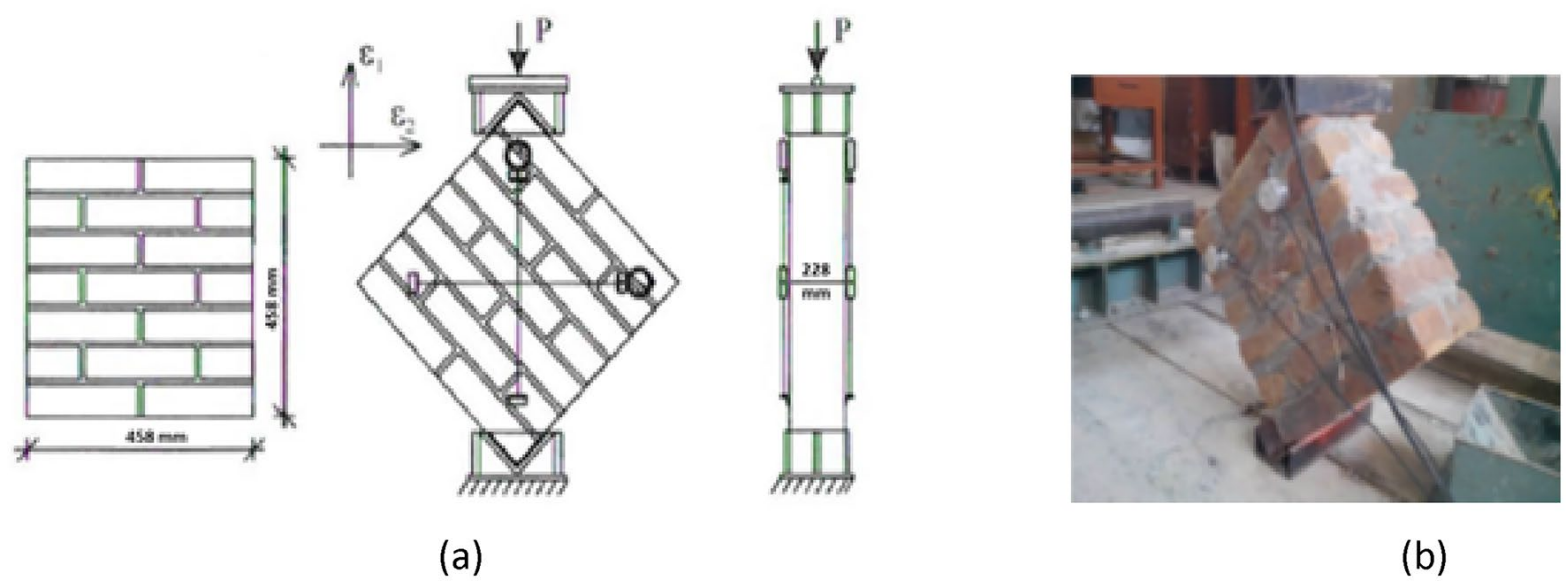
Diagonal compression test.

The RlLEM^49^ defines the test method for determining the capacity of masonry when subjected to diagonal cracking. The test procedure, presented in Fig. 9, comprised of putting a square wall section to diagonal compression by means of steel shoes (loading plates) at two opposite corners. The loading plates/shoes should be one tenth (1/10th) of the length of the side, using ASTM E519^53^ Specification. Uniform load was applied in uniform intervals by means of a vertical actuator in force control environment.

The test was conducted on ten specimens of size 458 × 458 × 229 mm. Monotonic force was applied and both the horizontal and vertical displacements were calculated on the two main sides of specimens (Fig. 9). The results are provided in Table 5. The approximated failure load of the 7 samples is equal to 6810 N and resulting the average diagonal compressive strength equal to 0.045 N/mm^2^.

**Table 5:** Diagonal compressive strength of specimens.

S. no.	Specimen type	Strength (MPa)	Average	Standard Deviation
1	Unconfined	0.043	0.045	0.0042
2		0.044		
3		0.049		
4		0.051		
5		0.039		
6		0.045		
7		0.04		

### Tests outputs

**Figure 10:**
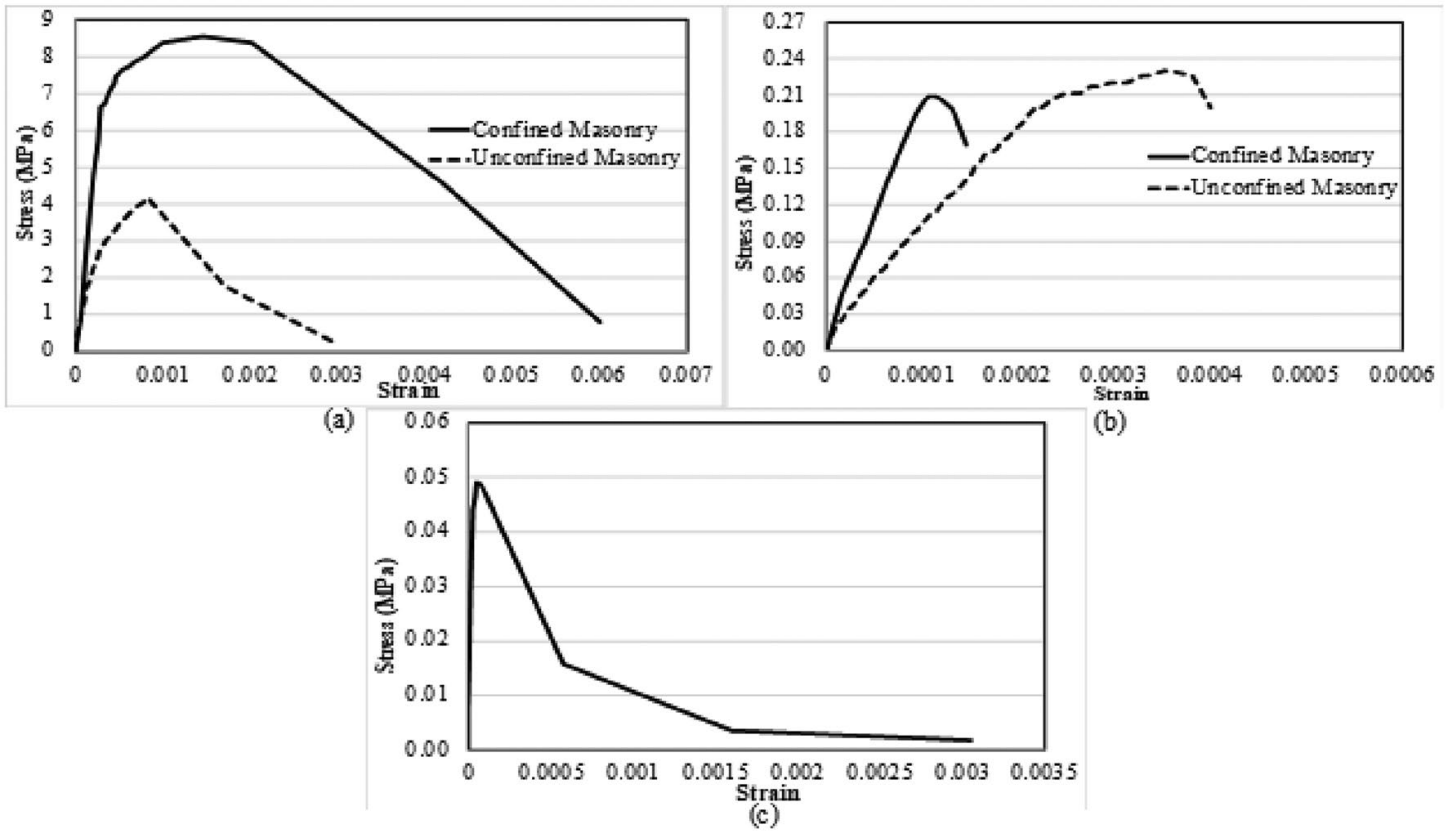
Stress–strain curves for (**a**) compression, (**b**) tension and (**c**) diagonal compression specimens.

Test results of masonry prisms (in the form of stress–strain curves) were utilized as inputs in the model. These comprise of elastic and plastic properties of unconfined samples. For this reason, an average compressive strength value of 4.15 MPa was used. This average value is obtained from tests performed on the group of five specimens. The elastic modulus and compressive behavior (inelastic) were evaluated from stress–strain curve and then further modified and applied in the succeeding FE based numerical model (Fig. 10a and b). Similar practice was followed in diagonal compression (DC) specimens and an average stress–strain curve with 0.045 MPa diagonal compressive strength was employed. Here, results from the horizontal LVDT were used for modeling the tension part of material model, as presented in Fig. 10c. Moreover, extension of horizontal diagonal in DC test, attaining 10% of the ultimate strength, was opted as inelastic displacement while describing the damage behavior of cohesive contact. By allowing 1.5 m gauge length, inelastic deformations in damage progress of prisms calculated 2 mm, respectively. Additionally, mortar (with type I cement i.e., ordinary Portland cement) was tested for determining its strength in compression. The mean value of mortar strength was 2.1 MPa. This value indicated the initiation of damage in the normal direction. The test results that were adopted for the model definition will be described in the subsequent section.

## Numerical modeling

### Material constitutive relationships

Numerous proposed models e.g., Giordano et al.^54^, Lemos^55^, Lofti and Shing^56,57^, Lourenco^58^, Lourenco et al.^26,59^, Page^60^ and Pegon and Anthoine^61^ have been opted for macro, meso and micro modeling methods, counting smeared- and discrete-crack models, damage- and plasticity-based models. In simplified micro-modeling (i.e., meso modeling) method, all component materials with discrete properties, are modelled autonomously. Separate material models for units, and interface are used. Material tests performed on masonry specimens^62^ are utilized in describing the model. The thorough explanation is presented here.

#### Concrete damage plasticity

To model the masonry unit, the “3D-Nonlinear Cementitious Material2” model provided in the commercially available program Atena 3D^63^ was employed. It is chosen here, because of its computational efficiency, and accurate in depicting the local response and failure pattern. The above-mentioned model is basically generated to estimate the response of concrete and similar materials with quasi-brittle nature like masonry, rock etc. Tensile cracking or compressive crushing are the key failure modes defined in the model. The damages due to tensile and compressive stresses by micro and macro cracks can be captured distinctly in this model. The compressive hardening and tensile softening characteristics of the brick units are defined by parabolic and exponential laws respectively, see Fig. 14. The properties opted for the model description are given in Tables 6 and 7. It is to mention here that the poison’s ratio for the units was taken from literature^50,51,63^ and was not determined experimentally in this study.

**Table 6:** Material properties of unit and interface.

Topology	Contact—Joint					Brick–element				
Material	3D Interface					3D Non-Linear Cementitious2				
Properties	*K*_*nn*_ (MN/m^3^)	*K*_*tt*_ (MN/m^3^)	*f*_*t*_ (MPa)	*c* (MPa)	Ф	*E* (GPa)	*G*_*F*_ (N/m)	*ν*	*f*_*t*_ (MPa)	*f*_*c*_ (MPa)
Values	350,000	120,000	0.40	0.52	0.33	23.65	39.67	0.2	1.46	14.40

**Table 7:** Properties of steel plates.

Topology	Steel plates		
Material	3D elastic isotropic		
Properties	*E* (GPa)	*ν*	*ρ* (MPa)
Values	400	0.3	0.02

#### Interface element

Normally, cohesive properties refer to the separation of the surface/edges of possible cracks. This idea of cohesive region was introduced by Dugdale^64^. Barenblatt^65^ taken this idea into account for fracture modeling of brittle materials. Needleman^66^ predicted that these cohesive elements are partly valuable when the interface is weak in comparison to the connecting materials. The application of model is very large to investigate the failure modes of many materials^67^, especially in cases when the interface properties and structural reliability is of interest. Coulomb-friction model was used for modeling the interface element to be serve as masonry joint between the units, see Fig. 15. The parameters of interface model are explained in section “Interface failure characterization" and the values are provided in the Table 6, where c and φ values are obtained from Table 2. However, the value of interface tensile strength (f_t_) is calculated by the method provided by Cervenka et al.^63^. The K_tt_, K_nn_ represent the elastic shear and normal stiffness respectively. Normally for interface elements with zero thickness, these stiffness values signify a high significance number. It is recommended^63^ to estimate the stiffness values using the formulas k_nn_ = E/t and k_tt_ = G/t, where t is the length of finite element used, and E and G is minimal elastic modulus and shear modulus respectively of the adjacent material. In addition to that, two added stiffness values represented in Fig. 15 as K_tt min_ and K_nn min_, are opted just for the sake of numerical reasons so that subsequent to the failure of the element, the global system of equations preserves its positive definiteness. Hypothetically, after the failure of interface its stiffness must be zero, which would cause the global stiffness to be indefinite. Therefore, K_tt min_ and K_nn min_ should be about 1/1000th of the initial stiffnesses.

### Geometry and meshing

The meshing details along with the loading conditions of samples are presented in Fig. 12. Bricks are modeled as isoparametric solid element named as “CCIsoBrick” with mesh size of 50 mm (2 in). The mesh was basically comprised of interface and continuum elements to characterize the joint and unit respectively. It is normally believed that a fine mesh will give more accurate detailing of cracks and other stress concentrations and hence more precise results. Research works relating to the high loading rates have adopted the FE meshes as small as 2–3 mm^68–70^. However, finer meshes might not essentially depicts the actual results while modeling the brittle materials^71–76^. The generation of great quantity of elements can cause problems (numerical instability) exclusively in the crack-propagation process, since the number of cracks increases with each iteration due to the increase of the Gauss points. Hence, a sensitivity analysis of mesh was done to decide the best-fit size of mesh appropriate for masonry assembly integrating units and interface.

**Figure 11:**
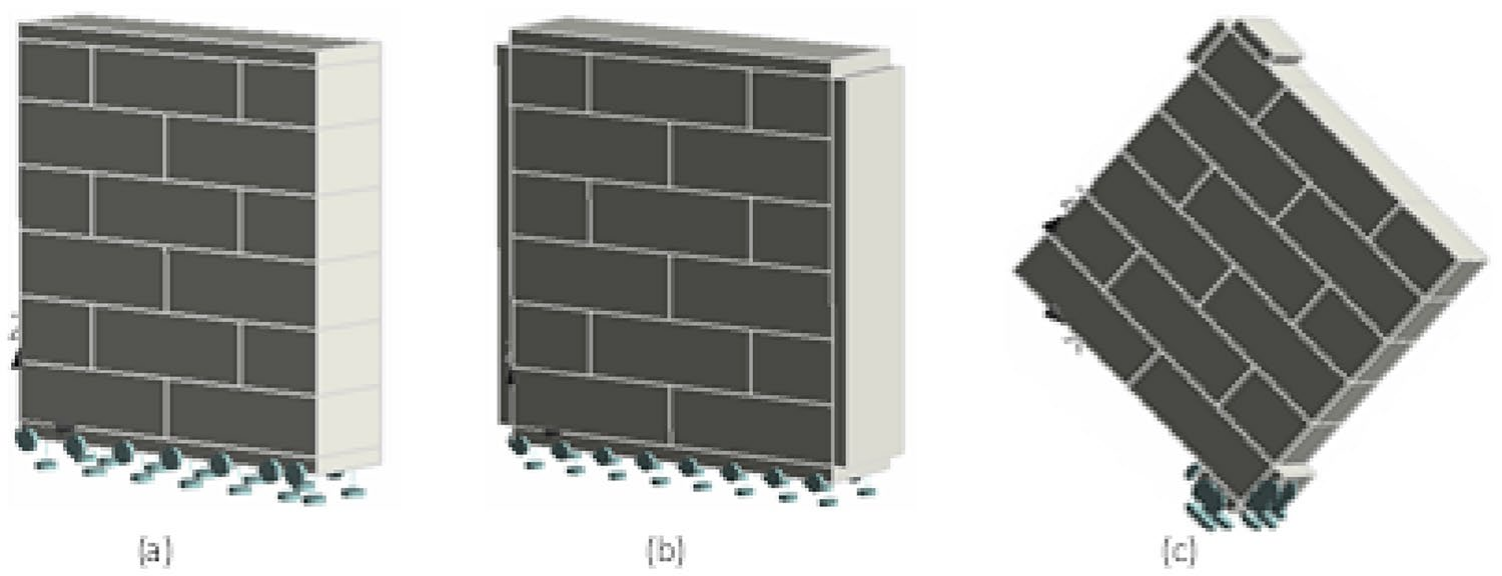
Geometry and boundary conditions of (**a**) unconfined, (**b**) confined, and (**c**) diagonally loaded prisms.

**Figure 12:**
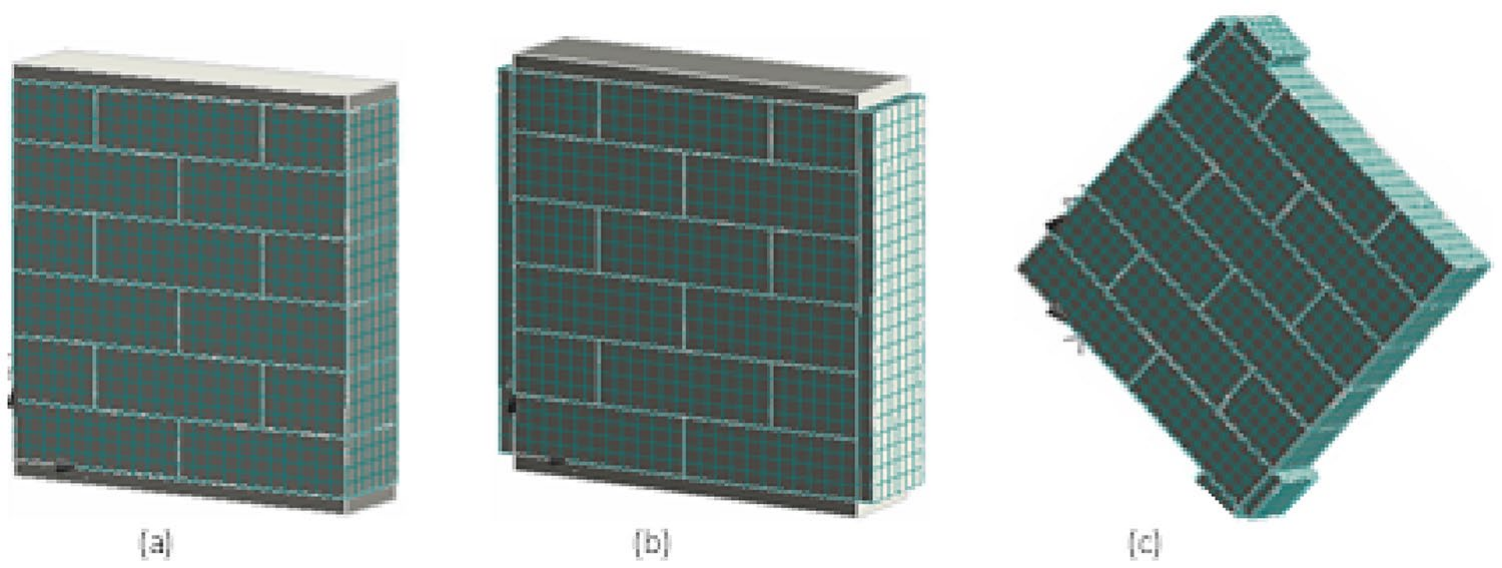
Meshing details of (**a**) unconfined, (**b**) confined, and (**c**) diagonally loaded prisms.

**Figure 13:**
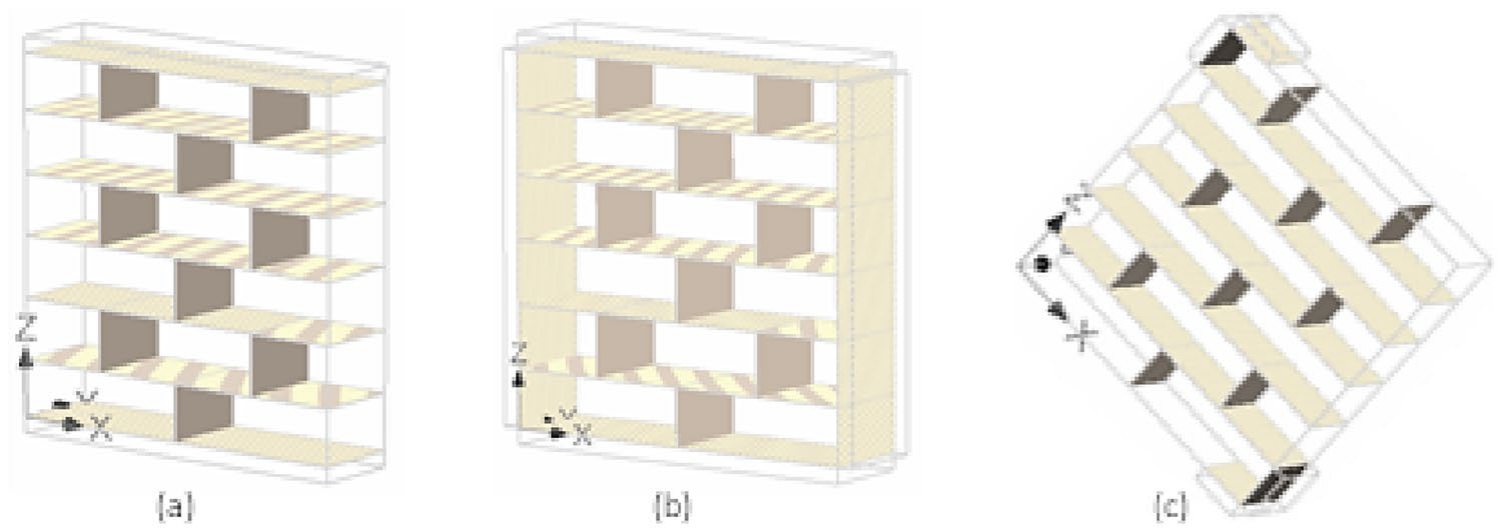
Surface-based interface detailing of (**a**) unconfined, (**b**) confined, and (**c**) diagonally loaded prisms.

Displacement control loading environment was used where all the models were tested by applying specified displacement to the steel plate, at the specimen’s top. Due to the great quantity of finite elements involved in the approach, thus, an eight-node 3D solid brick isoparametric element, integrated by Gauss integration were selected for modeling the brick units. Linear interpolation is used with 4 × 4 Gauss integration system. The model assumes a variable normal strain with a persistent shear strain. The specimen geometry, boundary conditions and distinct interface elements are presented in Figs. 11, 12 and 13, respectively. The brick unit size was 229 × 114 × 76 mm (9 × 4½ × 3 in) and the mortar thickness was taken zero. In unconfined samples, steel plates of 25.4 mm (1-in) width were used at top and bottom of the specimen for uniform distribution of stresses. The vertical load (tension/compression) in the form of displacement were applied to the top plate however the bottom plate was made fixed to match the exact experimental conditions. In case of confined samples, two additional steel plates were modeled (of same properties and cross section) at both sides of samples and were restrained against any in-plane lateral movement. As explained previously in experimental testing program, the plates in numerical model were given a very small inside lateral displacement (i.e. 0.01 mm) in the first step of load application and was not increased in the subsequent steps, in order to make sure that the plates should remain intact and resist the lateral in-plane displacement within the sample to offer a full lateral confinement in the in-plane direction. This very small initial value of confining load (in the form of displacement) was assigned to incorporate the effect of experimental tests where nut and bolt mechanism was adopted to join both the lateral plates for lateral confining effects. Finally, yet mechanical response and fracture mechanics of diagonally loaded sample is complicated as compared to confined and unconfined prisms, generally the unit properties govern vertically however the interface properties governs laterally. Moreover, crack opening response was given to the interface element through coulomb friction model, to define the relation among various surfaces, as shown in Fig. 13.

## FE model, results and discussion

### Model inputs

#### Fracture-plastic model parameters

**Figure 14:**
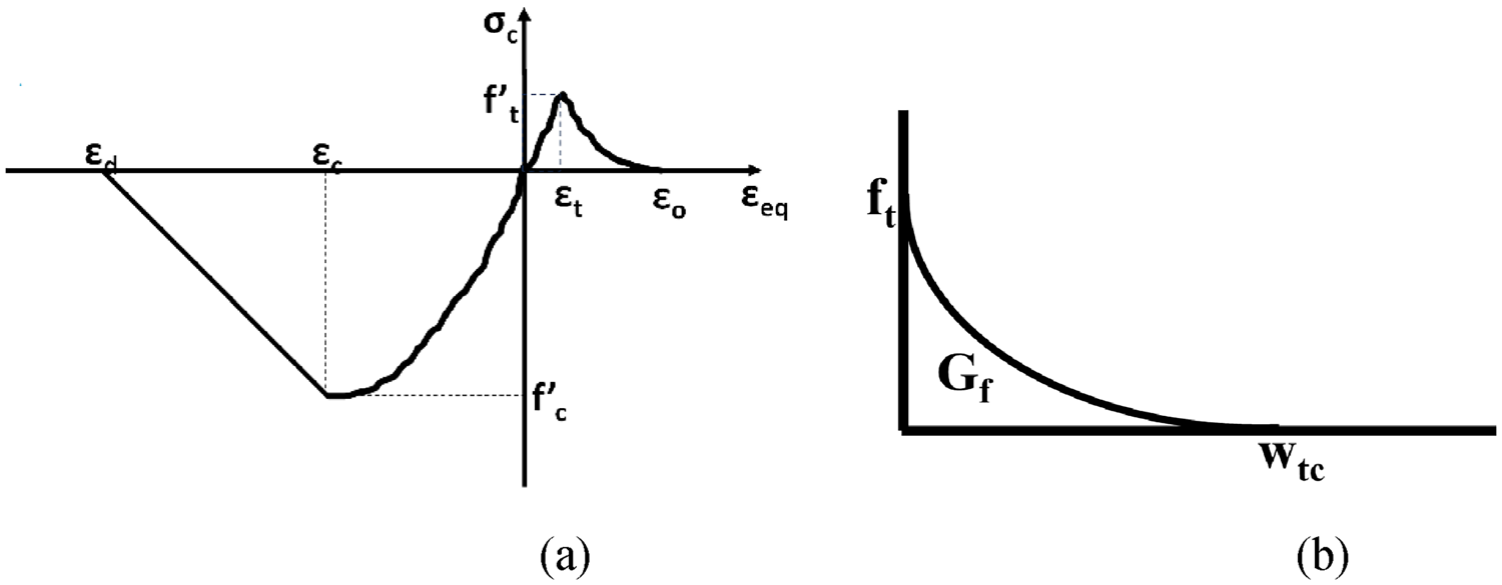
(**a**) Complete stress–strain behavior and (**b**) tensile softening of Brick unit^63^.

The brick unit is modeled with “3D Non-linear Cementitious 2” model provided in the commercially available software ATENA. Its complete material behavior in both tension and compression is depicted by a single stress–strain curve (Fig. 14a). Parabolic constitutive law is employed for the compressive hardening behavior, however exponential constitutive law is adopted for describing the tensile softening behavior of the unit, see Fig. 14. The material parameters employed are presented in Table 6.

#### Joints cohesive behavior parameters

**Figure 15:**
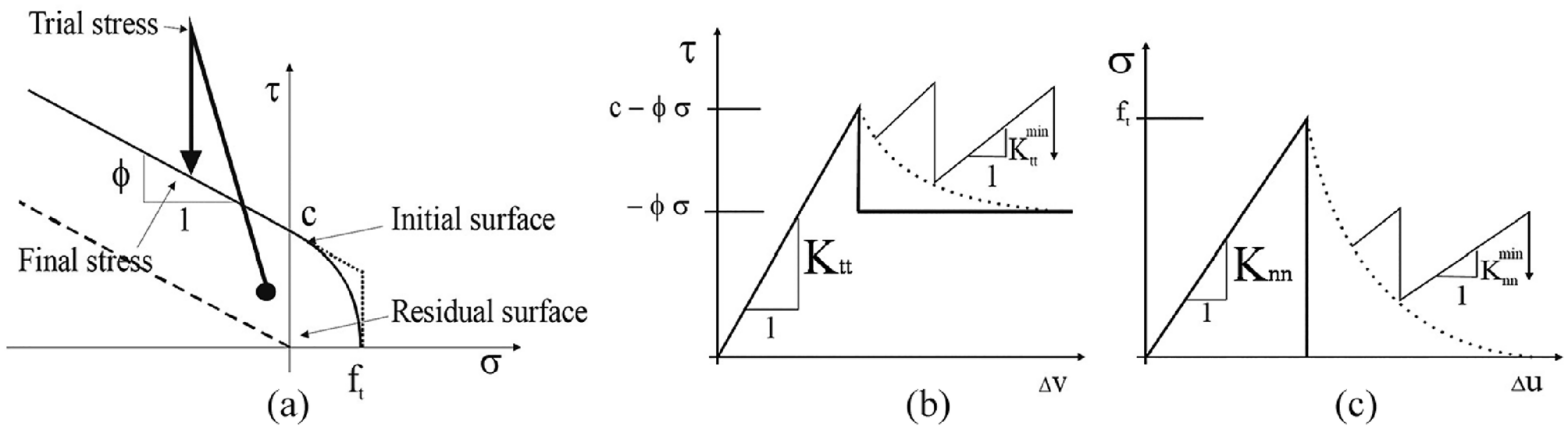
(**a**) Failure surface for interface element, (**b**) behavior of interface element in shear, and (**c**) in tension^63^.

Mortar joint is the solitary cause against shear forces by bond resistance by the bed joint. Thus, the mortar strength was opted for describing the response of masonry specimens in cohesion. As discussed in section “Interface element”, mortar joints were simulated with an interface element using coulomb-friction principle, see Fig. 15. Various parameters required for describing the model i.e., cohesion, tangential stiffness, and friction angle were taken from shear testing of the triplet and are explained in section “Interface failure characterization”. But, the bond strength (in tension) test of duplets (masonry prisms) was used for evaluating the tensile strength of interface. The properties used for modeling the interface element are also presented in the Table 6. The interface element was simulated as “hard contact” in the perpendicular direction while a “frictional” in the parallel direction.

### Model outputs

**Figure 16:**
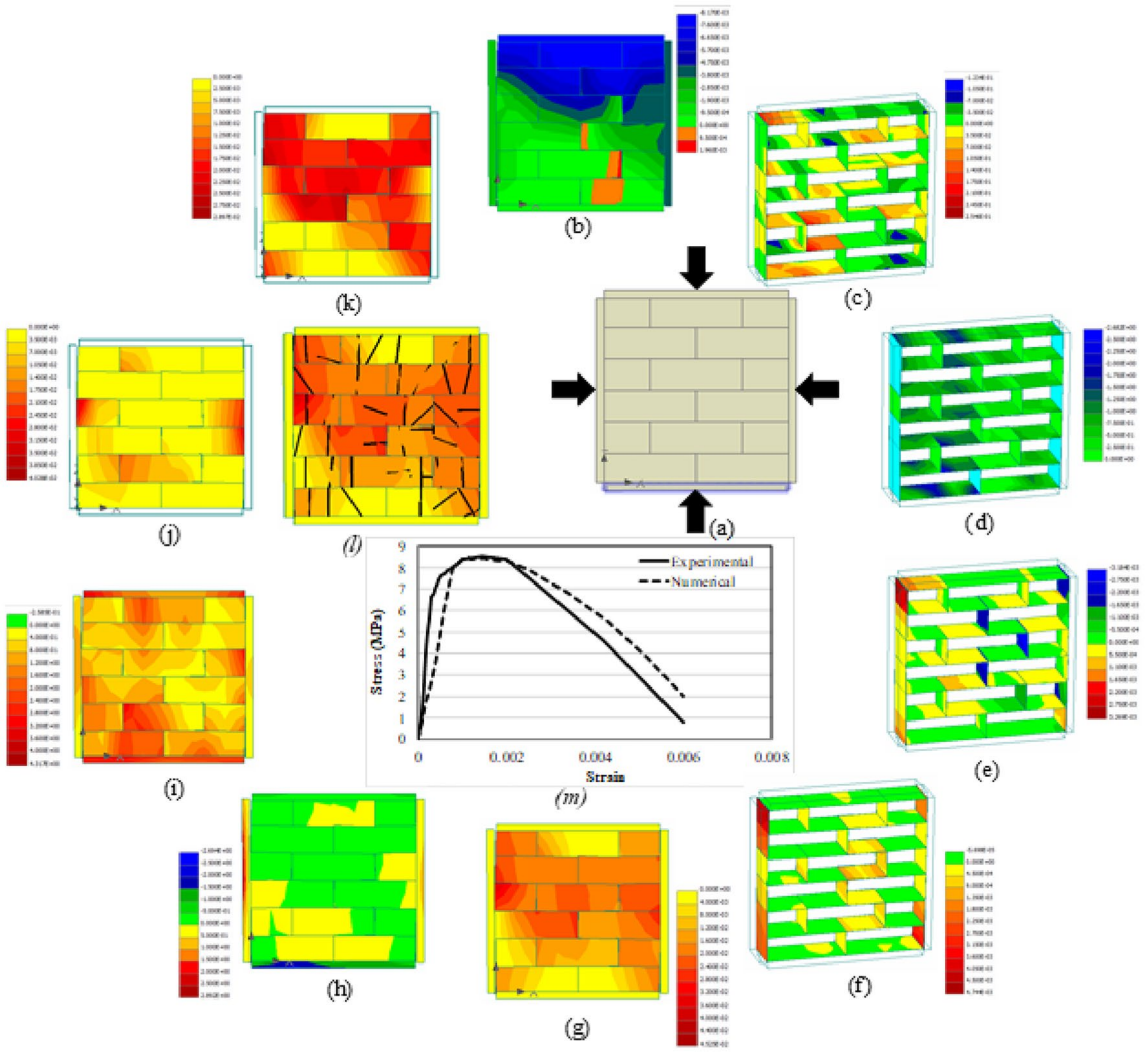
CC load–displacement curve and other stress and strain contours.

**Figure 17:**
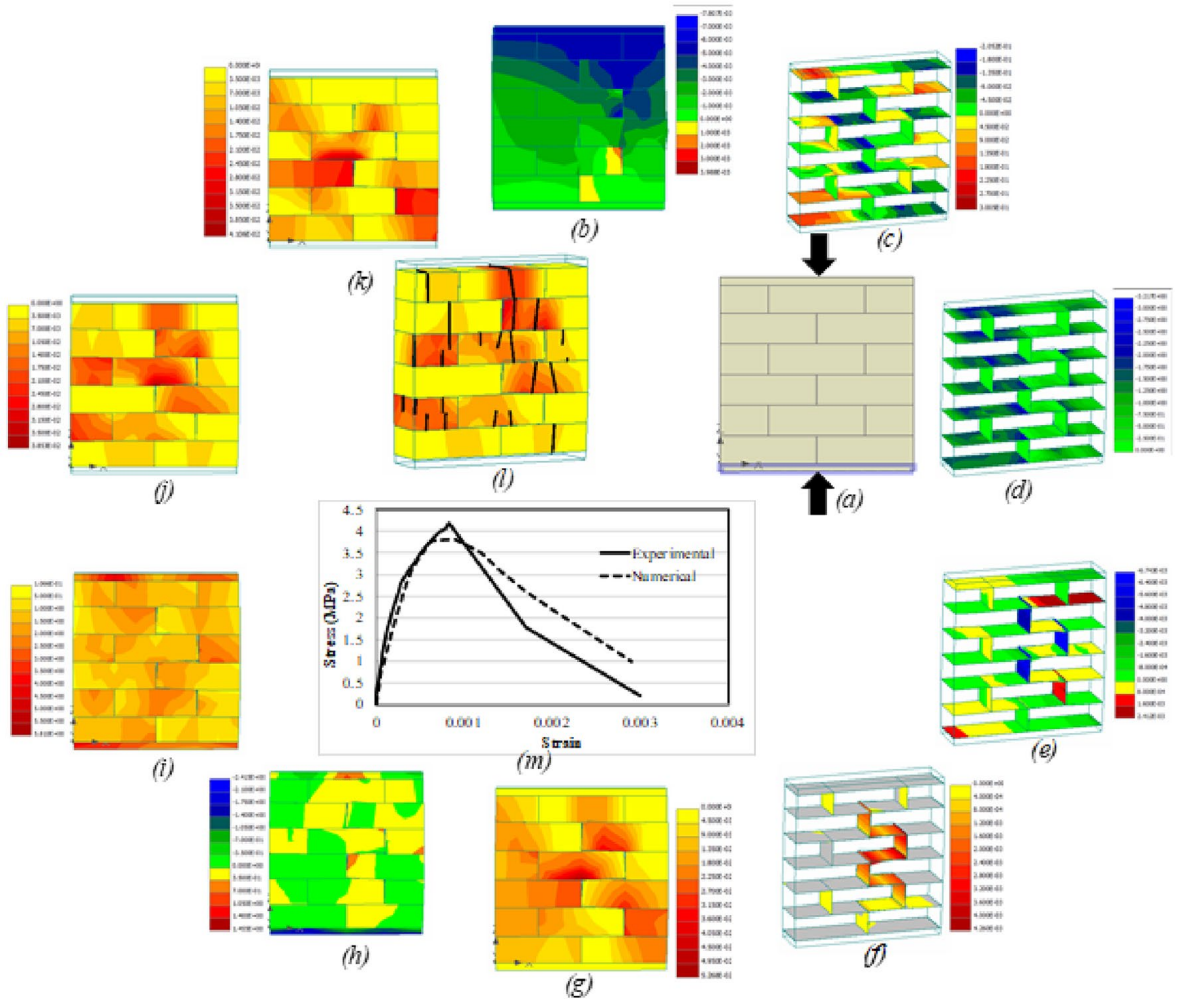
UC load–displacement curve and other stress and strain contours.

**Figure 18:**
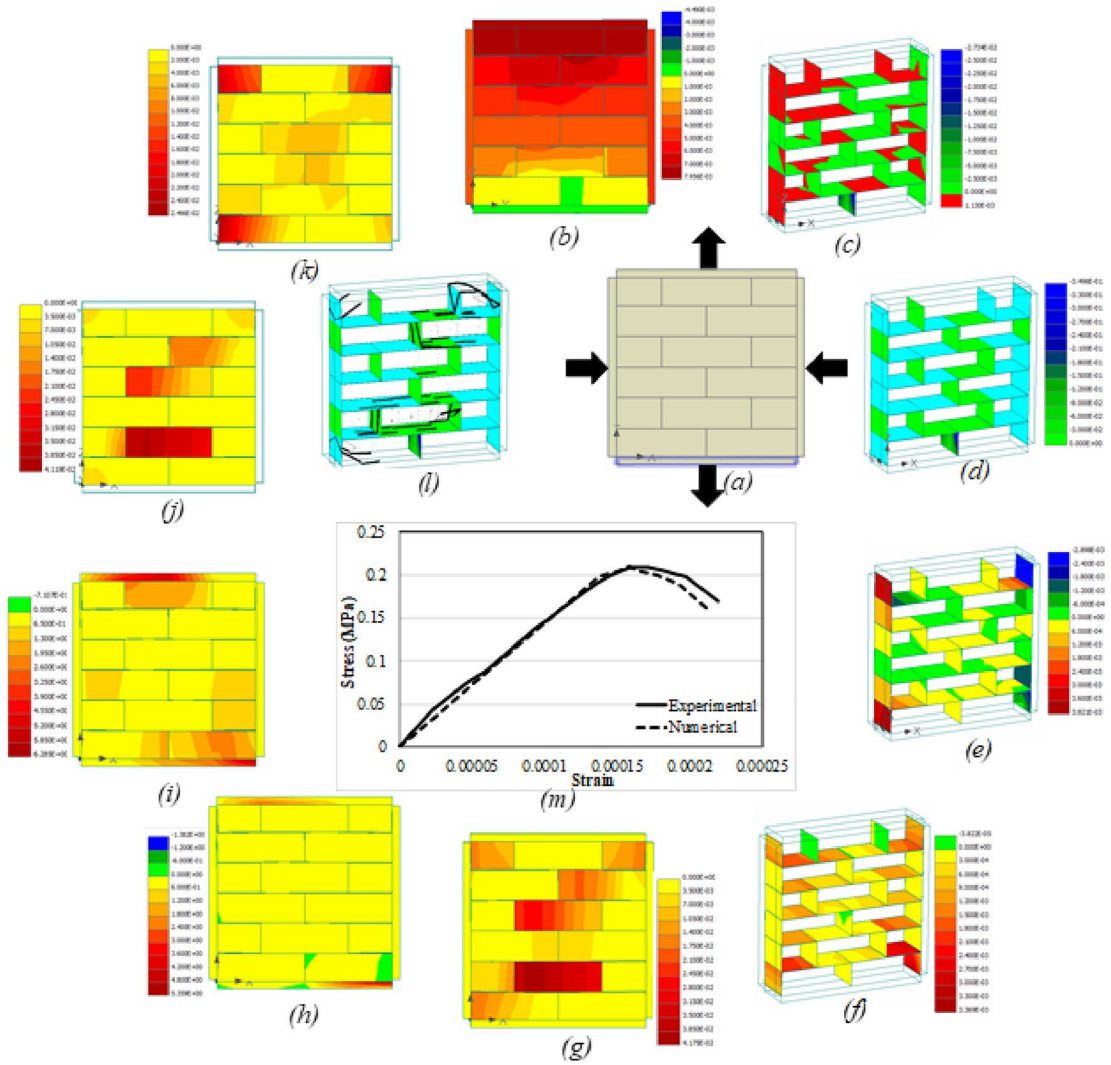
CT load–displacement curve and other Stress and Strain Contours.

**Figure 19:**
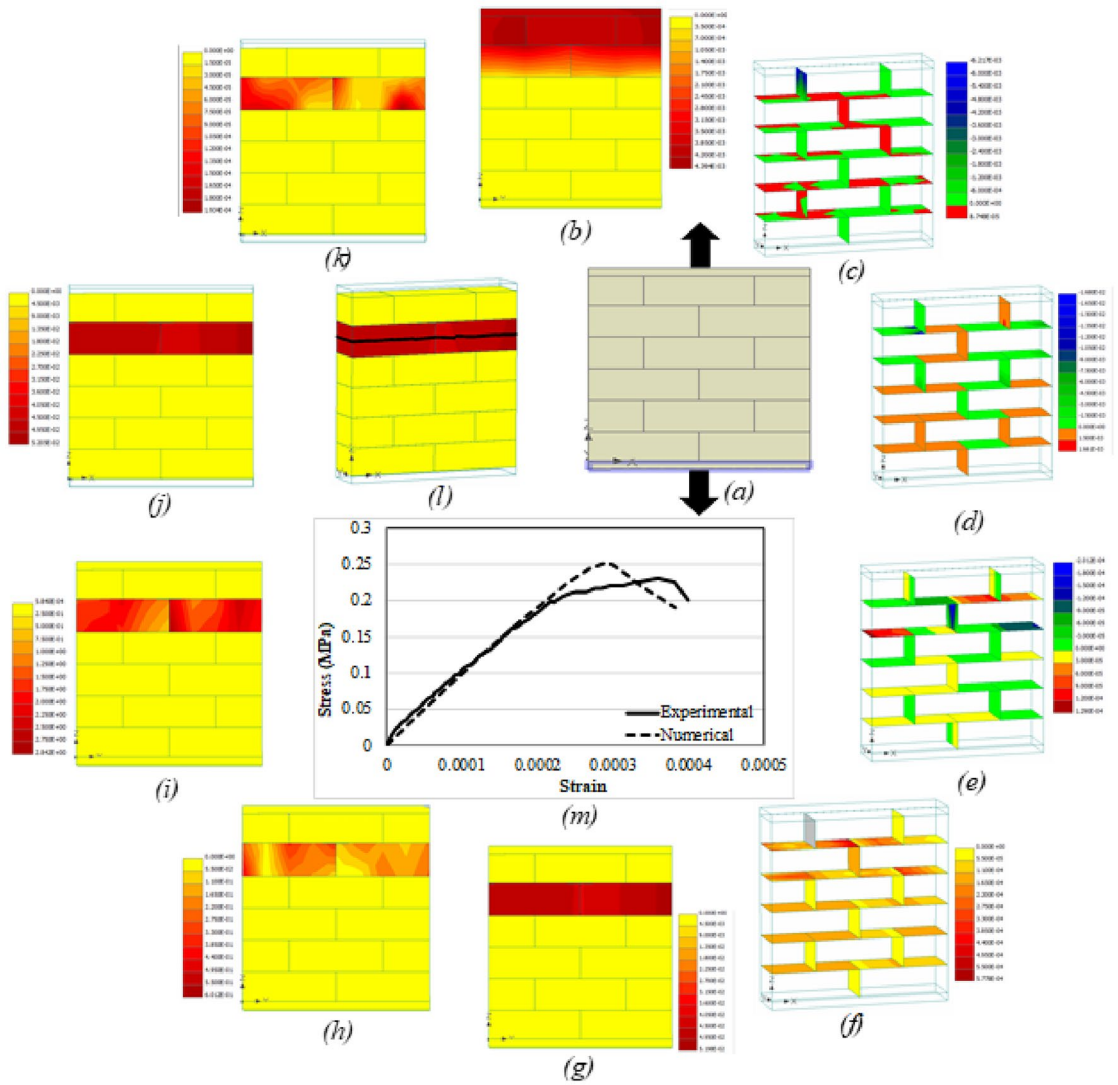
UT load–displacement curve and other stress and strain contours.

**Figure 20:**
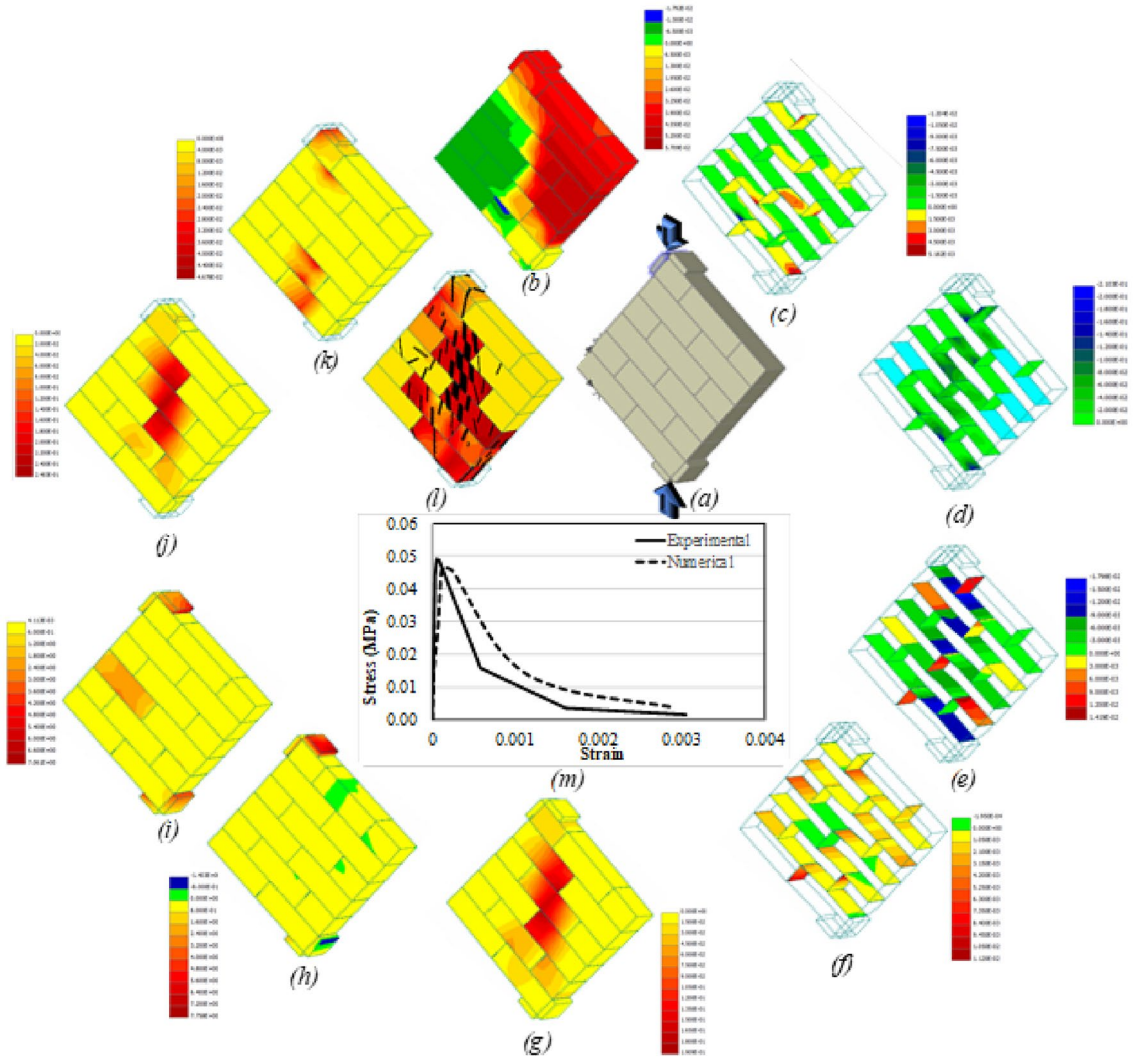
DC load–displacement curve and other stress and strain contours.

Apart from the load–displacement curve, other parameters that were studied included vertical displacement, interface shear (σT_1_) and normal (σN) stresses, interface shear (Dv_1_) and normal (Du) displacements, max. principal strain and stress, von misses stress, max. principal fracture strain (MPFS), principal plastic strain (PPS) and the crack pattern. The reason for selecting these parameters was to have a complete description of the behavior of masonry under various kind of loading and boundary conditions. Another reason was that till date, no one has investigated these parameters and their interrelation and interdependency for better understanding of the response of masonry. Figures 16a–m, 17, 18, 19 and 20a–m presents the CC, UC, CT, UT and DC prisms, respectively. The numerical analysis results are in good conformity with the experimental results. All the output parameters will be discussed one by one for all the five conditions to better understand the difference in terms of behavior and load distribution. It should be noted here that all the parameters (*b*–*l*) presented in Figs. 16, 17, 18, 19 and 20 are plotted at the ultimate damage point i.e., when the test was completed, and the results obtained in the last step are plotted to show the final result. Since it was already discussed previously that the bottom plate was fixed and compressive displacement was applied only at the top plate therefore (Figs. 16, 17, 18 and 19a), the vertical displacement (in both confined and unconfined specimen) started from the top layer of the specimen and gradually decreases towards the bottom of the specimen. The results quite satisfactorily depict the actual behavior of the specimen in which the compression displacement is maximum in the layer where load is applied and gradually decreases towards the layer that is resting on a fixed surface (see Figs. 16b and 17b). The confinement seems to have a role as well to keep the vertical displacement towards the middle of the specimen in the form of an arch, which is somewhat different than the unconfined prism. In case of tension members, the confinement tends to help the whole prism to act as the assembly in taking the tensile load opposite to the unconfined prism where the top layer seems to have almost all the vertical displacement, thus indicating the separation of top layer from the rest due to bond failure/failure of the adjacent layer of units (see Figs. 18b and 19b). Since the top layer was attached to the steel plate therefore the effect of steels stiffness is transferred to the top layer and the failure happens at the second layer. Very similar failure was observed in experimental testing as well in the case of UT test. The vertical displacement of diagonal specimen is very clearly showing the stress concentration at the middle of the specimen as can be seen easily that the displacement in one side is quite larger than the other side, thus resulting in the failure along the middle vertical line as discussed before (Fig. 20b).

The interface stresses (normal and shear) and displacements (normal and shear) also indicative of the type of failure occurring in that specimen. Due to the confinement effect the joint displacements and stresses are smaller in CC prism (Fig. 16c–f) as compared to the unconfined one. Given the fact that strength of confined samples is double as compared to the unconfined ones, still the stress concentration at interfaces is lesser in confined samples whereas the UC samples (Fig. 17c–f) were failed due to the excessive displacement and stress concentration at the interfaces (i.e., both head and bed joints). By comparing the Fig. 16d and f with the Fig. 17d and f, it becomes easier to understand that interface normal displacement and normal stress are more concentrated at the middle center of unconfined sample as compared to the confined sample where the stresses were distributed along the whole assembly primarily due to the confining effect. In case of tension members (confined and unconfined), the interface normal stress particularly indicates the concentration of stresses in the top layer of interface in case of UT (Fig. 19c–f) while in the CT prism (Fig. 18c–f) the stresses seem to be more distributed because of the lateral confinement effects. In general, lateral confinement enhances the performance of specimen in compression and reduces the performance in tension, as above-mentioned figures clearly depict the distribution of stresses among the interface and units in confined compression as compared to unconfined compression, where the stresses are more concentrated at the interface thus causing the interface to govern the response completely with much involvement of the units. However, in the case of tension, since a very small confinement displacement was applied parallel to bed joint, which causes the tensile effect in bed joints even before the application of vertical tensile force, and therefore, shows less strength then the unconfined tensile specimen. In the case of diagonal compression specimen, the interface displacements and stresses move along the straight line between the two ends vertically, thus confirming the splitting phenomena discussed previously (Fig. 20c–f).

The maximum principal strain and stress even more clearly shows the confinement effects in both the compression and tensile loading cases. The UC sample (Fig. 17g, h) shows the stress concentration more obvious at the middle as compared to the confined one (Fig. 16g, h) where the stress was distributed throughout the sample. Similarly in case of UT (Fig. 19g, h) the principal strain and stress appear to be concentrated on the top layer of interface (i.e., bed joint) and the adjacent unit layer, however in confinement case (Fig. 18g, h), the effect of confinement can be seen where stresses/strains are seen in the middle of the prism due to lateral confinement. The deformed shape of the assembly also highlights the confinement effect, as in unconfined samples the distance among the units keeps on increasing after the failure of interface, unlike the confined samples where the lateral confinement keeps intact the assembly even after the failure. For diagonal compression specimen (Fig. 20g, h), the principal strain diagram is well in agreement with the previous results however the principal stress diagram does not show any stress concentration at the face of the specimen.

Von mises is another criterion to see the stress concentration and section utilization under the external loading conditions. The UC sample (Fig. 17i) shows that most part of the material yields/fails under the loading, however the corners and sides of the samples have taken less load as compared to the middle center of the assembly. Contrary to the CC sample (Fig. 16i), the yielding/failure is seen in almost all the assembly. A similar pattern like before is seen in UT (Fig. 19i) and all stresses are concentrated to the top layer of interface and the adjacent unit layer. And more stress distribution is seen in the CT (Fig. 18i) case where the sample is subjected to the lateral compression as well. In summary, von-mises stresses reveals that in unconfined compression, the stresses are more concentrated at the middle part of the masonry sample, however, the confinement makes these stresses to distribute evenly in the whole prism thus utilizing the strength of complete sample instead of a portion of it. However, in the tension case, the von mises stresses also shows a concentration of stress in the pure tension case, whereas, the confinement induces some preliminary stresses that changes the typical stress pattern of tensile stresses. In case of diagonal compression specimen, the von mises stresses also didn’t appear at the surface of the specimen just like principal stresses and therefore no conclusion can be drawn from this (Fig. 20i) as well.

Figures 16j, 17, 18 and 19j presents the dispersal of the MPFS throughout the prism, the arrangement of fracture strains confirms what was first observed for the max. Principal stresses for CC, UC, CT and UT specimens. However, the difference is much obvious in case of confined compression as compared to unconfined one, where the fracture strain is more concentrated in the middle of the specimen of the unconfined case in contrast to the confined case where no concentration is noticed and thus the whole assembly is subjected to more or less similar kind of stresses. In case of diagonal compression specimen (Fig. 20j), the MPFS gives a very clear indication of the stress concentration at the middle vertical diagonal just like the actual tested results. Similarly, Figs. 16k, 17, 18, 19 and 20k presents the dispersal of the PPS and its arrangement confirms what was observed for the max. Principal strain for all the five cases considered i.e., CC, UC, CT, UT and DC specimen.

The crack pattern is also presented for all the samples and is in total agreement with the experimental testing results. The crack pattern of CC prism (Fig. 16l) shows the crack dispersion in the whole sample owing to the fact that the lateral confinement kept the sample intact even after the cracking of units and failure of interface, thus pushing the uncracked units to participate in the load bearing mechanism thus increasing the overall capacity of the assembly. UC samples (Fig. 17l) on the other hand shows the cracks at the failure point and where the cracks start to propagate from the top to bottom of the sample. The crack pattern quite differs from the confined sample. In CT case, cracks mostly occurred at the interfaces, primarily because of the lateral compression and then due to the applied tensile loading. The cracking is quite dispersed in CT case as compared to the UT case, where the cracking is concentrated at the top interface and unit layer (Fig. 18l and Fig. 19l). In case of diagonal compression specimen, the crack pattern clearly indicates that due to the stress concentration at the vertical diagonal of the specimen, the cracks generated at propagated through this diagonal thus causing the splitting of the specimen at the middle.

It is noteworthy that the stress applied normal to the bed joints, due to vertical loads and gravity, sustains a great amount of slip displacement after the adhesive bond failure. Thus, causing a ductile failure and huge amount of absorbed energy in the prisms loaded axially. The numerically obtained stiffness is quite similar to the experimental one, though, the shear strength obtained in the numerical analysis is fractionally greater than the experimental one (Figs. 16m, 17, 18, 19 and 20m). Moreover, as presented in Figs. 16, 17, 18, 19 and 20, the interface between surfaces and gap experiences the maximum stress. Figure 20 presents load–deformation response of the upright diagonal of the diagonal compression specimens. Unconfined specimens performed better than confined ones in terms of deformation capacity. The model predicted the response of diagonal compression specimen fairly well. The distribution of stresses is nearly equal and thus it was revealed that the maximum portion of load is sustained by the sample’s diagonal portion. As anticipated, both sides of the model do not contribute in taking the load and deformation.

## Summary and conclusions

The main objective of this study was to evaluate the lateral in-plane confinement effect on the mechanical properties as well as ductility of brick masonry samples, and to use these results for numerical modeling in order to study the in-depth behavior of these masonry prisms. The experimental test results revealed that behavior (deformation capacity, strength and failure mode) of unconfined masonry is quite different than that of confined assemblies. To better understand the response of masonry assemblies, a modified micro modeling method has been adopted to generate the FE model of assemblies. Different input parameters like uniaxial stress–strain behavior, elastic and inelastic masonry properties, yielding and failure criteria etc., were used in the numerical model obtained from various experimental tests. Units and joints were homogenized as a smeared material into enhanced units with modified dimensions, and were modeled using damage plasticity model. Coulomb friction behavior was adopted for the interface between the enhance units, as a gap element with zero thickness. Damage initiation was counted based on mortar strength. Damage progression, crack width at zero load, was classified as a plastic displacement. The material properties of masonry were taken from the compression and diagonal shear tests. It is clear from the results that the behavior determined through the numerical analysis are promising with those of the tested specimens. The following conclusions are based on the findings of this study.

1. The lateral confinement significantly enhances the compressive strength of brick masonry. The average ultimate strength was increased by as much as 103%. The lateral confinement manages to reinforce the fragile mortar joints (in the lateral in-plane direction) resulting in more resistance to the crack propagation. Therefore, confining the masonry prism in lateral in-plane direction simply improves the compressive-, shear- and diagonal compressive strength of assemblies.
2. The average strain at fracture and hence the ductility of the confined masonry sample was also increased by 101% as compared to the unconfined one. The ductility is very important parameter especially when the earthquake forces are to be considered. Hence the lateral confinement of the masonry simply enhances its ductility properties and makes it more resilient to the seismic forces.
3. The lateral confinement has a negative effect on the axial tensile strength of the masonry, and the tensile strength decreases by confining laterally, although very small.
4. The confinement effect may decrease the workmanship effect on the brick masonry response. Since the workmanship quality has a massive effect on the properties of masonry structures. From Table 3 the trend that become apparent is that the standard deviation in ultimate strength decreases significantly due to the lateral in-plane confinement of the prisms.
5. The numerical model also captured these variances with the least error. The proposed model can be successfully used to model masonry walls and structures under both the confined and unconfined conditions. The ultimate strength, strain at ultimate strength, strain hardening and strain softening curves, strain at rupture etc. are very well depicted by the model with great accuracy.

## Data Availability

The data set used/or analyzed during the current study available from the corresponding author on reasonable request.
